# Oridonin Attenuates Myocardial Ischemia/Reperfusion Injury via Downregulating Oxidative Stress and NLRP3 Inflammasome Pathway in Mice

**DOI:** 10.1155/2020/7395187

**Published:** 2020-05-30

**Authors:** Chuanghong Lu, Chuanbin Chen, Ang Chen, Yunjiao Wu, Jianlin Wen, Feng Huang, Zhiyu Zeng

**Affiliations:** ^1^Department of Cardiology, The First Affiliated Hospital of Guangxi Medical University, Nanning, Guangxi 530021, China; ^2^Guangxi Key Laboratory of Precision Medicine in Cardio-Cerebrovascular Diseases Control and Prevention, Guangxi Clinical Research Center for Cardio-Cerebrovascular Diseases, Nanning, Guangxi 530021, China; ^3^Department of Geriatric Cardiology, The First Affiliated Hospital of Guangxi Medical University, Nanning, Guangxi 530021, China

## Abstract

Oridonin (ORI), the major pharmacological component extracted from a traditional Chinese medicine, possesses a beneficial effect on myocardial ischemia/reperfusion (I/R) injury. However, the underlying molecular mechanism by which ORI effects take place is not completely known. Thus, whether ORI works via downregulating oxidative stress and nod-like receptor protein-3 (NLRP3) inflammasome pathway was investigated in this study. Mice underwent surgery to induce myocardial I/R injury, and some were administered ORI (10 mg/kg/day) pretreatment, while others were not. The myocardial enzymes' levels, infarct area, and inflammatory injury were measured. The activation situation of oxidative stress and NLRP3 inflammasome was also detected. We found that ORI pretreatment significantly alleviated CK-MB and cTnI levels and infarct size induced by I/R. ORI mitigated the inflammatory injury by decreasing the pathological damage and lowering TNF-*α*, IL-1*β*, and IL-18 levels. Moreover, the SOD1 and eNOS levels were significantly increased by ORI, while MDA and iNOS levels were relatively decreased. The oxidative stress was reversed using ORI pretreatment. Importantly, NLRP3 inflammasome pathway was also inhibited by ORI, as reflected by the lower protein levels of NLRP3, caspase-1, and IL-1*β*. In conclusion, ORI alleviates myocardial injury induced by I/R via inhibiting the oxidative stress and NLRP3 inflammasome pathway.

## 1. Introduction

It is known that myocardial ischemia/reperfusion (I/R) injury will appear when the partially or completely occlusive coronary artery is strategically reopened in acute myocardial infarction (AMI) patients [1, 2]. During reperfusion, the ischemic myocardium can only be partially salvaged, and further damages will flood in [3, 4]. Abundant radical oxygen species (ROS) and proinflammatory mediators are released from the injured mitochondria to tissue fluids, which will lead to severe inflammatory injury [5, 6]. Over the past few decades, various pharmacological interventions have been used to improve the survival of myocardial tissues after artificial reperfusion [7, 8]. However, the clinical effects are not very satisfactory. Thus, more effective drugs are still required for treatment.

Nod-like receptor protein-3 (NLRP3) inflammasome is an assembled molecular complex consisting of NLRP3, apoptosis-associated speck-like protein containing CARD (ASC), and cysteinyl aspartate specific proteinase (caspase-1) [9]. Due to the myocardial ischemia injury and mitochondrial damage, NLRP3 inflammasome is subsequently activated and increased gradually within 6–24 h [10, 11]. Furthermore, upon its activation, proIL-1*β* and proIL-18 are converted to active forms, which aggravate the inflammatory injury and increase the infarct area [12]. Studies have demonstrated that inhibiting the activity of NLRP3 inflammasome directly or indirectly is beneficial in terms of decreasing the inflammatory injury and infarct area [13, 14]. NLRP3 inflammasome is a popular therapeutic target in treating myocardial I/R injury.

The extracts from natural herbs or plants, as demonstrated in previous studies, help in decreasing the cardiovascular diseases' risk [15, 16]. Oridonin (ORI) is the major pharmacological component of rabdosia rubescens [17, 18], which has been used for a long time as a Chinese medicinal herb for some inflammatory diseases. During the past few years, ORI has also been demonstrated to have strong antioxidative and NLRP3 inhibiting properties [19–21], besides its popular antitumour activity [17, 22]. Moreover, Gong et al. reported that ORI promoted the survival rate of myocardial cells after hypoxia process by downregulating apoptosis and autophagy [23]. Zhang et al. showed that ORI alleviated myocardial I/R injury by regulating the energy and metabolism of amino acids in rats [24]. Thus, ORI is safe and effective and seems to have a potentially remedial effect on myocardial I/R injury.

However, the mechanisms by which ORI attenuates myocardial I/R injury are not completely known. Thus, we explored the effects of ORI on oxidative stress and NLRP3 inflammasome pathway in a mouse model, trying to illuminate the mechanisms of ORI in attenuating myocardial injury induced by I/R.

## 2. Materials and Methods

### 2.1. Study Subjects

All male C57BL/6 mice (7-8 weeks old, 18–22 g), supplied from the Experimental Animal Center of Guangxi Medical University, were raised in a 12 h light/dark cycle room with normal water and feed. Room temperature was maintained at 20°C–25°C, and humidity was 50%–60%. All of the animal experiments and related laboratory operations in this study were approved by the Ethics Committee of The First Affiliated Hospital of Guangxi Medical University.

### 2.2. Grouping and Pretreatment

Sixty mice were divided randomly into four groups (15 mice/group): (1) sham group: mice underwent the surgical procedure except for the left anterior descending (LAD) coronary artery ligation; (2) I/R group: the LAD of mice in this group was carefully ligated for 30 min followed by a 24 h reperfusion as described previously [13]; (3) I/R + ORI group: mice were administered ORI (10 mg/kg, Sigma-Aldrich, USA) daily, which was dissolved in 0.1 ml vehicle solution (1% dimethyl sulfoxide (DMSO, Sigma-Aldrich, USA) diluted by normal saline, using intraperitoneal injection for seven days before the surgical operation [24]; (4) ORI group: mice were injected with an equal ORI solution in the same way and subjected to the sham operation. As a control, mice in the sham and I/R groups were administered an equal amount of 0.1 ml vehicle solution.

### 2.3. Myocardial I/R Model Establishment

The model establishment was performed as described in a previous study [25]. The mouse was anesthetized intraperitoneally using sodium pentobarbital (50 mg/kg) and was then ventilated endotracheally with a minivent mouse ventilator (type 845, Harvard Apparatus, Germany). The chest was carefully opened on the left side of the fourth or fifth intercostal space. In addition, the LAD was firmly ligated with 7-0 silk sutures for 30 min. Subsequently, the myocardium turned to be white, and an arc-like ST segment elevation was detected by electrocardiogram (ECG). The above phenomena indicated that the model was established successfully. After the ligature was released, it was followed by 24 h reperfusion. Finally, the mouse was anesthetized again and sacrificed for the following experiments.

### 2.4. ELISA for Myocardial Enzymes and Inflammatory and Oxidative Stress Markers

After 24 h of reperfusion, majority of the mice were anesthetized again, and the blood specimens were collected and centrifuged (3000 g, 4°C) for 15 min. The serum samples were isolated and preserved. Following the operating guidelines of manufacturers, cTnI, CK-MB, TNF-*α*, IL-1*β*, and IL-18 (all kits purchased from CUSABIO, China) levels were detected using corresponding ELISA kits.

### 2.5. 2,3,5-Triphenyltetrazolium Chloride (TTC) Staining

After 24 h of reperfusion, some of the mice were sacrificed for TTC staining. The hearts were frozen for 15 min and sliced into transverse sections (1-2 mm thickness). The slices were incubated with 2% TTC solution (Sigma-Aldrich, USA) for about 15 min and fixed well in 4% paraformaldehyde (Solarbio, China) for about 24 h. Under the observation, the infarct area was stained white, and the noninfarct area was stained red. The infarct size was exhibited as the percentage of myocardia infarct area accounting for the left ventricular area.

### 2.6. Hematoxylin and Eosin (H&E) Staining

Mice were sacrificed, and the myocardial tissues were fixed in 4% paraformaldehyde. According to the standard procedures, the samples were well embedded in paraffin, sectioned into slides, dewaxed, and stained using H&E dye. Finally, all the slides were pictured with the use of an optical microscope (CKX41, Olympus, Japan). Moreover, the injury condition was scored as described [26]: 0: without damage; (1): cells arranged roughly in order with mildly necrosis and edema; (2): cells arranged slightly disordered with moderate local necrosis and swelling; (3): cells arranged disordered with severe necrosis and inflammatory infiltration; (4): cells arranged extremely disordered with greatly severe diffuse necrosis and inflammatory infiltration.

### 2.7. Western Blot

Total proteins were extracted from the left ventricular precordial tissue (100 mg in average) using RIPA lysis (Solarbio, China) plus phenylmethyl sulfonylfuoride (PMSF). The lysate was drawn and centrifuged (12000 g, 4°C) for 5–10 min. The supernatant concentration was measured using BCA test kit (Beyotime, China) and was then boiled for about 5 min. Equal protein samples were separated using different densities of sodium dodecyl sulfate polyacrylamide gel electrophoresis (SDS-PAGE, Solarbio, China) and then transferred to the polyvinylidene fluoride (PVDF) membranes (EMD Millipore, USA). After 1 h of being blocked with 5% skimmed milk, all membranes were incubated overnight at 4°C with primary antibodies (1 : 500–1000): MDA (Abcam, USA), SOD1, eNOS, iNOS, NLRP3, ASC, pro-caspase-1, cleaved caspase-1, pro-IL-1*β*, IL-1*β*, and GAPDH (all purchased from CST, USA). Then, membranes were cleaned carefully and incubated with the horseradish peroxidase- (HRP-) conjugated secondary antibody (1 : 10000, Abcam, USA) at room temperature for 2 h. Finally, all blots were detected and quantified using the FluorChem FC3 system (ProteinSimple, USA).

### 2.8. Statistical Analysis

Each individual experiment was repeated at least three times. All data are exhibited as the mean ± standard deviation (SD) and were analyzed employing SPSS software (version 19.0, IBM, USA). The one-way analysis of variance (ANOVA) with the Bonferroni post hoc test was applied for data analysis. *P* < 0.05 was considered statistically significant.

## 3. Results

### 3.1. ORI Alleviated Myocardial Injury and Infarct Size in Response to I/R

**Figure 1:**
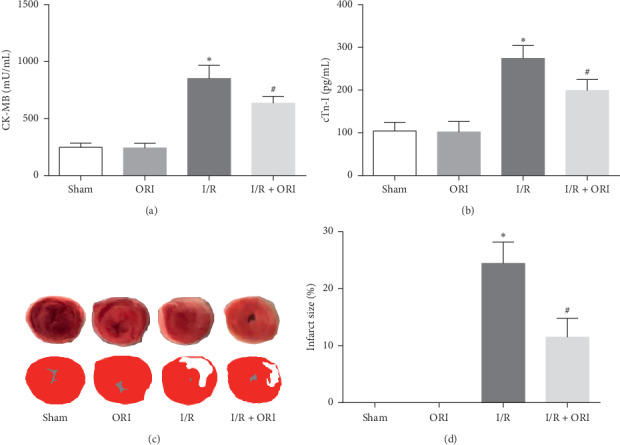
ORI decreased myocardial injury and infarct size induced by I/R. (a) Serum CK-MB level (*n* = 8). (b) Serum cTnI level (*n* = 8). (c) Representative images of 2,3,5-triphenyltetrazolium chloride (TTC) stained section. The infarct area is stained white, while the noninfarct area is stained red. (d) Infarct size (%), exhibited as the percentage of myocardia infarct area accounting for the left ventricular area. ^*∗*^*P* < 0.05, vs. sham group; ^#^*P* < 0.05, vs. I/R group.

To illustrate the protective effect of ORI on myocardial I/R injury, the myocardial enzyme markers, including CK-MB and cTnI, were determined in the serum by the ELISA method; the myocardial infarct area was also monitored using TTC staining. As shown in Figures 1(a) and 1(b), the I/R + ORI group exhibited significantly lower levels of CK-MB and cTnI in contrast to the I/R group (*P* < 0.05). Moreover, compared with the I/R group, the I/R + ORI group also showed obviously reduced infarct size (*P* < 0.05, 11.5% ± 3.3% vs. 24.4% ± 3.8%). Meanwhile, both the sham and ORI groups turned out to be normal with low CK-MB and cTnI levels and no infarct area.

### 3.2. ORI Mitigated Histopathological Damage and Inflammatory Injury Induced by I/R

**Figure 2:**
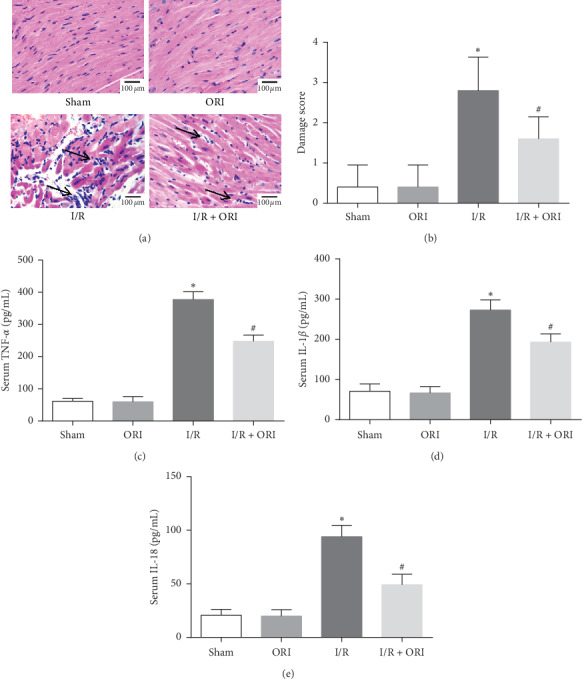
ORI reversed histopathological changes and inflammatory injury induced by I/R. (a) Representative pictures of Hematoxylin and Eosin (H&E) stained section (×200). The arrow indicates the presence of inflammatory cell infiltration. (b) Damage score. (c) Serum TNF-*α* level (*n* = 8). (d) Serum IL-1*β* level (*n* = 8). (e) Serum IL-18 level (*n* = 8). ^*∗*^*P* < 0.05, vs. sham group; ^#^*P* < 0.05, vs. I/R group.

To certificate the beneficial effect of ORI, we also investigated the myocardial histopathological changes using H&E staining and examined some inflammatory injury-related factors (TNF-*α*, IL-1*β*, and IL-18) using ELISA. As shown in Figure 2(a), myocardial cells of mice in the sham group and ORI group were arranged neatly with a complete cellular structure and were infiltrated with very few inflammatory cells in the stroma. However, cardiomyocytes in the I/R group were disorderly arranged with loose stroma and were flooded with substantial inflammatory cell infiltration as well as some necrotic cells. Importantly, compared with the I/R group, cardiomyocytes in the I/R + ORI group were arranged in order with mildly loose stroma and were surrounded with less inflammatory infiltration and necrotic cells. Moreover, as shown in Figures 2(c)–2(e), the I/R + ORI group indicated significantly lower levels of TNF-*α*, IL-1*β*, and IL-18 in contract to the I/R group (*P* < 0.05).

### 3.3. ORI Reversed I/R-Induced Oxidative Stress

**Figure 3:**
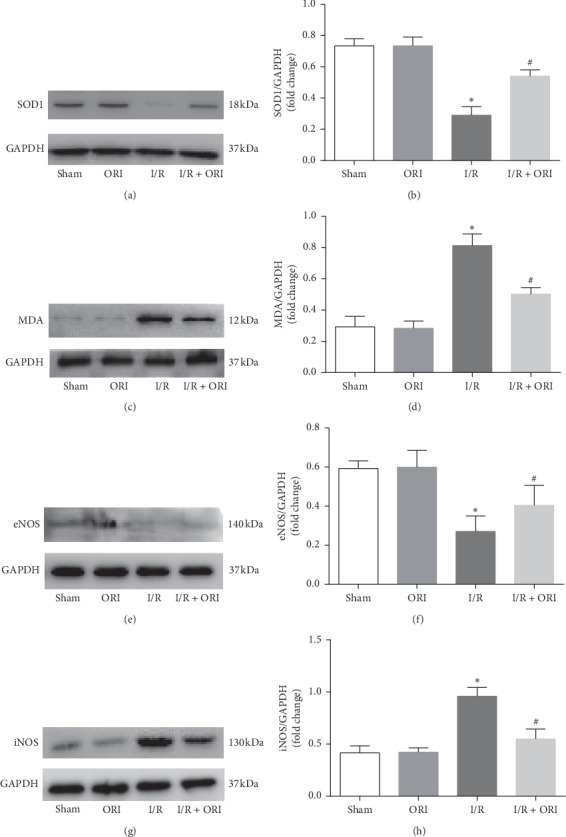
ORI alleviated I/R-induced oxidative stress. ((a), (c), (e), and (g)) Protein expression of superoxide dismutase1 (SOD1), malondialdehyde (MDA), endothelial nitric oxide synthase (eNOS), and inducible nitric oxide synthase (iNOS) in cardiac tissue detected by western blot and ((b), (d), (f), and (h)) their quantitative analyses. ^*∗*^*P* < 0.05, vs. sham group; ^#^*P* < 0.05, vs. I/R group.

In order to explore whether ORI influences oxidative stress induced by I/R, SOD, a major antioxidant enzyme, and MDA, a marker of degree of membrane peroxidation, were detected in the myocardial tissue by western blot. As pictured in Figures 3(a)–3(d), the SOD1 expression level was obviously decreased in I/R group (*P* < 0.05), while MDA level was significantly increased (*P* < 0.05); this adverse situation was reversed in the I/R + ORI group relative to the I/R group (*P* < 0.05). To further illustrate the antioxidation effects of ORI, endothelial nitric oxide synthetase (eNOS) and inducible nitric oxide synthetase (iNOS) were also determined in the myocardial tissues by western blot. As shown in Figures 3(e)–3(h), the situation of oxidative stress in response to I/R was significantly reversed in the I/R + ORI group relative to the I/R group, as reflected by the increased expression of helpful eNOS (*P* < 0.05) and the decline of damaging iNOS (*P* < 0.05).

### 3.4. ORI Inhibited NLRP3 Inflammasome Signaling Pathway

**Figure 4:**
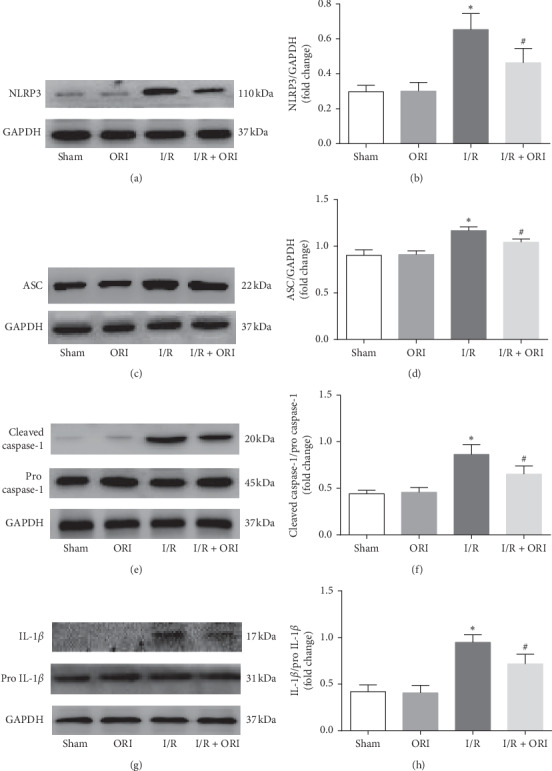
ORI inhibited NLRP3 inflammasome signaling pathway. ((a), (c), (e), and (g) Protein expression of NLRP3, ASC, caspase-1, and IL-1*β* in cardiac tissue detected by western blot and ((b), (d), (f), and (h)) their quantitative analyses. ^*∗*^*P* < 0.05, vs. sham group; ^#^*P* < 0.05, vs. I/R group.

To explore the activation situation of NLRP3 inflammasome induced by I/R with or without ORI treatment, the protein expression of the receptor NLRP3, the adaptor ASC, and the effector caspase-1 in the myocardial tissues were examined by western blot. As shown in Figures 4(a)–4(f), NLRP3, ASC, and cleaved caspase-1/pro-caspase-1 were apparently increased in I/R group relative to the sham group (*P* < 0.05). when, in contrast to the I/R group, however, this situation was significantly reversed in the I/R + ORI group in the presence of ORI pretreatment (*P* < 0.05). To further illustrate the NLRP3-inhibiting effect of ORI, IL-1*β*, the major inflammatory factor activated by NLRP3 inflammasome, was subsequently detected in the myocardial tissues. Correspondingly, as shown in Figures 4(g) and 4(h), IL-1*β* was also significantly decreased in the I/R + ORI group compared with the I/R group, as reflected by the lower level of IL-1*β*/pro-IL-1*β* (*P* < 0.05).

## 4. Discussion

In the present study, we investigate the underlying protective mechanisms of ORI on myocardial I/R injury. Our results show that ORI reduces the infarct size and pathological damage in cardiac tissues and alleviates the inflammatory injury induced by I/R. Importantly, ORI also downregulates the oxidative stress and NLRP3 inflammasome pathway.

ORI is the major bioactive substance extracted from rabdosia rubescens [18] and possesses potentially cardioprotective effects as mentioned before. In the present study, our results reveal that ORI reduces serum myocardial enzyme levels and infract size induced by I/R, which are basically consistent with the former study [24]. More importantly, we also found that the inflammatory injury induced by I/R is well reversed by ORI pretreatment, as reflected by the less pathological damage and lower TNF-*α*, IL-1*β*, and IL-18 levels (Figure 2). Also, it has been demonstrated that ORI alleviates vascular inflammation by decreasing inflammatory cytokine and endothelial adhesion molecules [27]. Taken together, ORI has an anti-inflammatory effect and alleviates myocardial injury induced by I/R. Furthermore, the molecular mechanisms by which ORI limits the inflammatory injury are further explored in the following sections.

During reperfusion, numerous radical oxygen species (ROS) and proinflammatory mediators are increasingly released from injured mitochondria [5, 6]. Meanwhile, oxidative stress is aggravated, as reflected by an imbalance between prooxidants and antioxidants, leading to an increase in inflammatory injury [28]. Over the past few decades, oxidative stress has been widely researched in myocardial I/R injury [5, 28]. Easing the oxidative stress level, as demonstrated in the previous study, is a considerable step in treating myocardial I/R injury [29, 30]. In our study, oxidative stress is well reversed by the ORI pretreatment as shown in Figure 3. Besides, ORI has also been reported to increase the antioxidase activities in a mouse model of aorta coarctation [31]. Therefore, we hold that ORI has an antioxidative effect and can treat myocardial I/R injury.

Followed with the increasing release of ROS and many other damaging factors, NLRP3 inflammasome is also subsequently activated and increased gradually within 6–24 h of reperfusion [10, 11]. During the past few decades, NLRP3 has been a widely concerned therapeutic target researched in the inflammatory injury induced by myocardial I/R. Several NLRP3 related inhibitors have been found or synthesized with good inhibitory activity and have shown to alleviate myocardial damage in animal model of myocardial I/R injury [32–35]. Importantly, ORI, extracted from a traditional Chinese medicine, has also been demonstrated to firmly bind to the cysteine 279 of NLRP3 and has a strong inhibiting effect [19]. In our previous experiments as shown in Figures 2(d) and 2(e), serum IL-1*β* and IL-18, which are activated by NLRP3 inflammasome in response to I/R [12], are obviously decreased by ORI pretreatment. Then, we subsequently found that NLRP3 inflammasome signaling pathway is significantly inhibited by ORI, with lower levels of NLRP3, caspase-1, and IL-1*β* expression (Figure 4). Not long ago, Yang et al. also reported that ORI alleviated carrageenan-induced pleurisy by inhibiting NLRP3 pathway in vivo [36]. Taken together, we tend to agree that ORI may mitigate myocardial injury via inhibiting the NLRP3 inflammasome activation, which might be one of the meaningful mechanisms underlying the therapeutic effect of ORI on myocardial I/R injury.

As demonstrated, this study clarifies the underlying protective mechanisms of ORI on myocardial I/R injury. However, there still exist some notable problems and limitations. First, this study focused on the protective effects of ORI pretreatment on myocardial I/R injury. To be closer to clinical practice, the effects of perioperative and postoperative treatment with ORI should be further investigated separately. Second, the oxidative stress level in this study was examined in some acceptable ways, but we did not detect the level of various ROS such as O_2_^−^, H_2_O_2_, and OH^−^. Third, our findings were based on animal experiments, and it would be better if in vitro experiments were performed. Finally, we did not employ NLRP3 inhibitors such as INF4E and OLT1177 or related siRNA in this study for further argumentation.

## 5. Conclusion

In summary, ORI salvages cardiomyocytes in danger and alleviates inflammatory injury induced by I/R. Importantly, the oxidative stress and NLRP3 inflammasome pathway are also downregulated by ORI pretreatment. Those current observations clarify the potential molecular mechanism by which ORI attenuates myocardial I/R injury. It is believed that the application of ORI would be beneficial for the prevention and therapy of myocardial I/R injury.

## Data Availability

The experimental data in this study can be obtained from the corresponding authors under reasonable requirement.
